# A new marine gastrotrich from the State of São Paulo (Brazil), with a key to species of *Pseudostomella* (Gastrotricha, Thaumastodermatidae)

**DOI:** 10.3897/zookeys.223.3975

**Published:** 2012-09-26

**Authors:** M. Antonio Todaro

**Affiliations:** 1 Department of Life Sciences, University of Modena and Reggio Emilia, via Campi, 213/D, 41125 Modena, Italy

**Keywords:** Gastrotrichs, Brazil, São Paulo, biodiversity, taxonomy, new species, key

## Abstract

In previous papers, faunistic and preliminary taxonomic data on the gastrotrich communities along the coastline of the Brazilian states of São Paulo and Rio de Janeiro were reported; among the over 40 records, the occurrence of several species new to science was highlighted. One of such new taxa is described here based on observation carried out on living and SEM prepared specimens. *Pseudostomella dolichopoda* **sp. n.** (Gastrotricha: Thaumastodermatidae) is the only species in the genus that attains 420 µm in total length, is covered by pentancres and possesses, among others, caudal pedicles up to 45 µm in length. Additional differences with co-generic taxa characterized by a pentancrous covering are discussed. Furthermore, a key to the described *Pseudostomella* species of the world based on easily discernible traits, visible in both living and formalin-fixed specimens, is provided.

## Introduction

The study is part of a larger research program aimed at shedding light on the diversity of marine invertebrates of the northern coasts of the State of São Paulo, Brazil (see Migotto and Tiago 1999). The results of this ambitious task will provide the necessary background for any future study meant at the assessment of the health of the marine environment and therefore at the sound management of its biota. In previous papers, faunistic and preliminary taxonomic data on the gastrotrich communities along the coastline comprised between Picinguaba to the north (at the Rio de Janeiro-São Paulo States border) and praia Preta e Choncas to the south were reported. Among the some 40 taxa found, the occurrence of several species new to science was highlighted (Todaro and Rocha 2004, 2005). One of such new taxa is described here, it is a macrodasyidan in the family Thaumastodermatidae. Beside the novelty, the new species bears also ecological significance in that it is highly represented throughout most of the investigated localities, constituting one of the most common and abundant interstitial meiofaunal taxon.

## Methods

Sandy sediment from the littoral and/or sublittoral site of 23 locations along the northern coasts of the State of São Paulo was collected during several field trips between 20 April and 3 May 2002. Littoral samples were taken during low tide, by digging three 30 cm-deep holes, ca 5 m apart from each other, at mid-water mark, and collecting the sand, about 500 ml, from the wall using a steel spoon. Sublittoral sand was taken by scooping the top sediment layers with a 500 ml plastic jar; sediment below 4 m water depth was collected by SCUBA diving. After collection, samples from each site were taken as soon as possible to the São Paulo University’s CEBIMar laboratory in São Sebastião. A general account on the visited locations including geographic coordinates and physical and chemical characteristics of the microhabitats are reported in Todaro and Rocha (2004).

**Figure 1.**
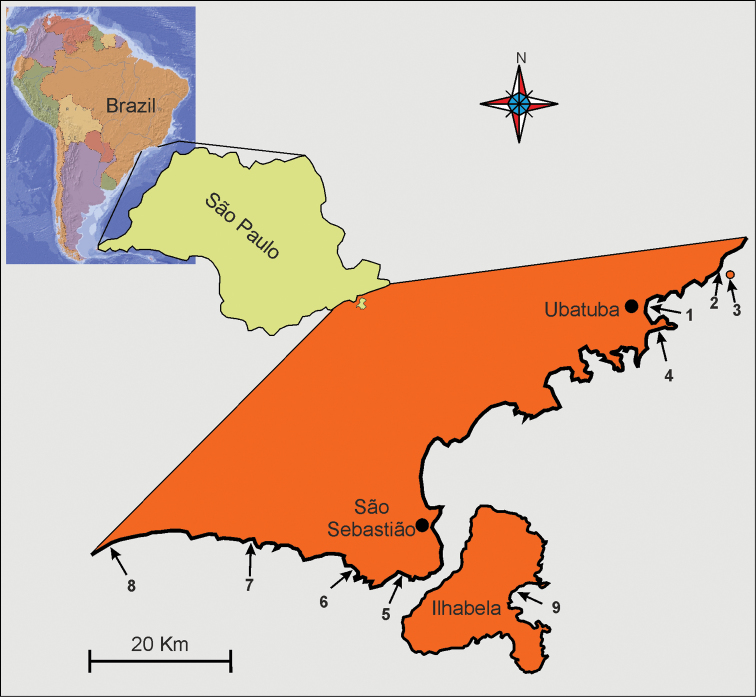
Localities along the cost of the State of São Paulo (Brazil) where *Pseudostomella dolichopoda* sp. n. was found. **1** praia Grande **2** praia Prumirim **3** Ilha do Prumirim **4** praia do Tenório (Ubatuba district) **5** praia de Guaecá **6** praia de Santiago **7** praia do Saí **8** praia Preta e Conchas(São Sebastião district) **9** praia de Castelhanos (Ilhabela island). For geographic coordinates and characteristics of the microhabitat see Todaro and Rocha (2004).

Using the same techniques, additional samples were taken in September 2003 from six beaches, four new and two already investigated (see Todaro and Rocha 2005); unless otherwise specified, data refer to the 2002 campaign. In Figure 1, the localities where the new species was found are reported. In the laboratory, the specimens were extracted daily with the narcotization-decantation technique using a 7% magnesium chloride solution within one week of collection. The supernatant was poured, without filtering, into 3.0-cm diameter plastic Petri dishes and scanned for gastrotrichs at 50  × under a Wild M8 stereomicroscope (see Todaro and Hummon 2008). Found gastrotrichs were mounted on glass slides and observed *in vivo* with Nomarski differential interference contrast optics using a Zeiss Axioscop 2 Plus microscope. During observation, the specimens were measured using an ocular micrometer and photographed with a Nikon Coolpix 995 digital camera (3.34 Mpixel). Some specimens were fixed overnight in a 1.0 M phosphate-buffered (pH 7.3) solution of paraformaldehyde, gluteraldehyde and picric acid, following Ermak and Eakin (1976), and stored for later SEM analysis. To this end, gastrotrichs were rinsed in 0.2 M cacodylate buffer, dehydrated through a graded ethanol series, critical point-dried using CO_2_, mounted on aluminium stubs, sputter coated with gold-palladium and observed with a Philips XL 30 scanning electron microscope at the author’s Institution.

The description of the new species follows the convention of Hummon et al. (1993), whereas the position of some morphological characteristics along the body are given in percentage units (U) of total body length measured from anterior to posterior.

Granulometric analysis of the substrata was carried out according to Todaro et al. (2006). Mean grain size, sorting coefficient, kurtosis, and skewness were calculated by a computerized program based on the equation of Seward-Thompson and Hail (1973).

Abbreviations are as follows: TL, total body length; PhL, pharynx length; PhIJ, pharyngeo-intestinal junction; TbA, adhesive tubes of the anterior series; TbDL, adhesive tubes of the dorsolateral series; TbL, adhesive tubes of the lateral series; TbVL, adhesive tubes of the ventrolateral series; TbP, adhesive tubes of the posterior series.

The rationale for the key to the ecological characteristics of the species, according to Hummon et al. (1992),is as follows: frequency of a species from among a sample series (i.e., frequency of a species in samples collected in any given sampling trip) - Sparse, found in less than 10% of samples; occasional, found in 10–30% of samples; common, found in 30–60% of samples; usual, found in more than 60% of samples.

Abundance of a species among other species of a sample - Rare, less than 1% of a sample; scarce, 3–5% of a sample; numerous, 10–20% of a sample (often a sub-dominant); prevalent, more than 30% of a sample (usually dominant or co-dominant).

## Taxonomic account

### Order Macrodasyida Remane, 1925 [Rao & Clausen, 1970]. Family Thaumastodermatidae Remane, 1927. Subfamily Thaumastodermatinae Remane, 1927. Genus *Pseudostomella* Swedmark, 1956

#### Pseudostomella dolichopoda sp. n.

urn:lsid:zoobank.org:act:89445CF5-147A-4B27-A403-0DD50268E442

http://species-id.net/wiki/Pseudostomella_dolichopoda

**Figure 2.**
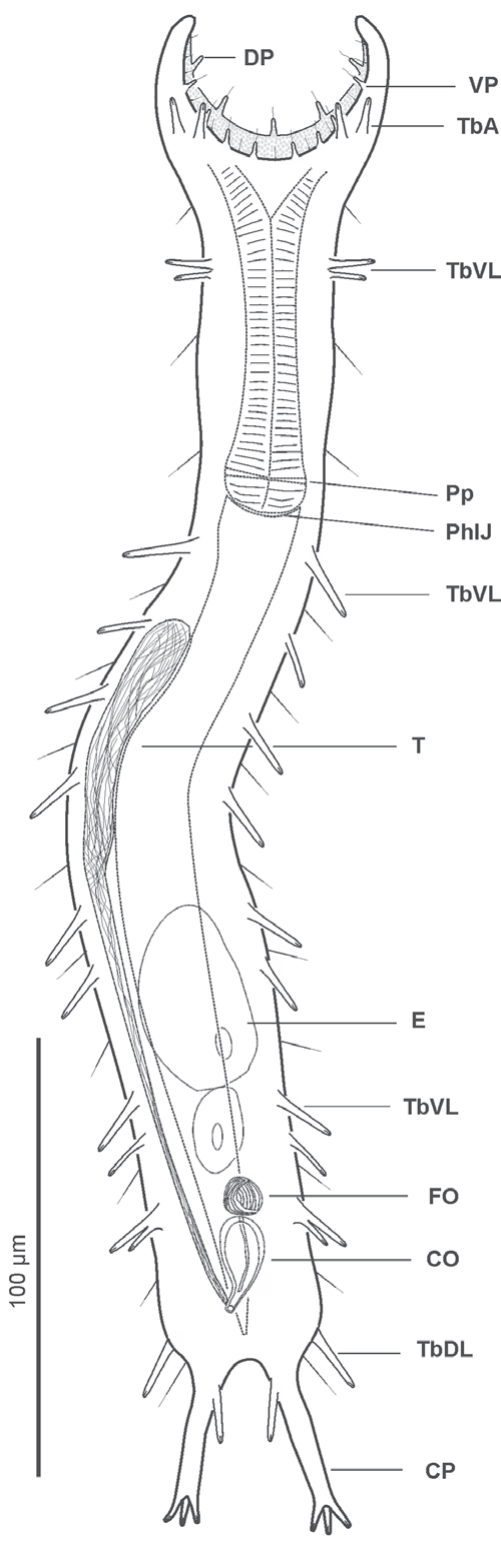
*Pseudostomella dolichopoda* sp. n. schematic drawing. Habitus as seen from the ventral side. **CO** caudal organ **CP** caudal pedicle **DP** dorsal papillae **E** egg **FO** frontal organ **PhIJ** pharyngeo-intestinal junction **Pp** pharyngeal pores **T** testicle **TbA** anterior adhesive tubes **TbDL** dorsolateral adhesive tubes **TbL** lateral adhesive tubes **TbVL** ventrolateral adhesive tubes **VP** ventral papillae.

Figures 2 [Fig F3] –4

##### Type locality.

Brazil, State of São Paulo, Praia Grande of Ubatuba (23°23'04.4"S; 45°03'49.9"W). At Mid-Water Mark, in fine (mean grain size, 0.160 mm ) moderately well sorted (sorting, 0.70 mm) siliceous sand, and at 1.5 m water depth in fine (mean grain size, 0.153 mm), moderately sorted (sorting, 0.54 mm), siliceous sand. Values of salinity, temperature and pH of the interstitial water at date of sampling 35.1 psu, 26.3° C and 7.92 respectively; from the same beach additional specimens were collected on 7 September 2003. Other location in the State - Ubatuba: praia Prumirim (**sl**=sublittoral) also in 2003, Ilha do Prumirim (2003, **l**=littoral), praia do Tenório (2003, **sl**); São Sebastio: praia de Guaecá (**sl**), praia de Santiago (**sl**), praia do Saí (**l, sl**), praia Preta e Conchas (**sl**); Ilhabela: praia de Castelhanos (**sl**) (see Fig. 1 and also Todaro and Rocha 2004 for additional details on these localities).

##### Type specimens.

**Figure 3. F3:**
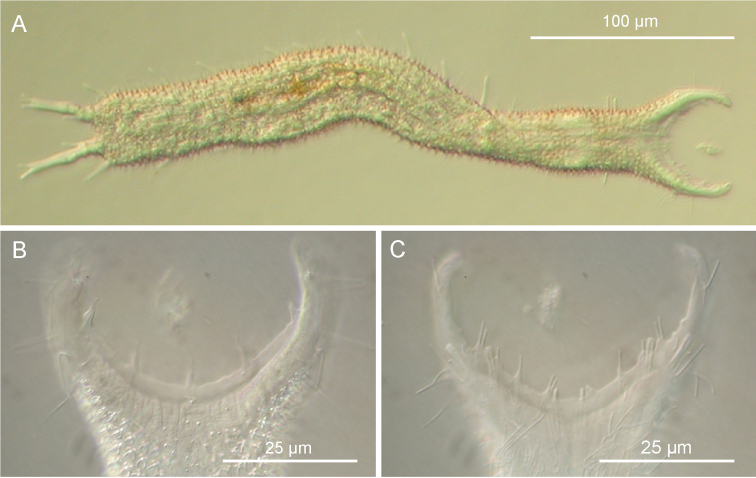
*Pseudostomella dolichopoda* sp. n. DIC photomicrographs. **A** habitus **B** close-up of the anterior region, dorsal view **C** Close-up of the anterior region, ventral view.

Holotype, the 358 μm long adult specimen shown in Figure 3.

**Figure 4.**
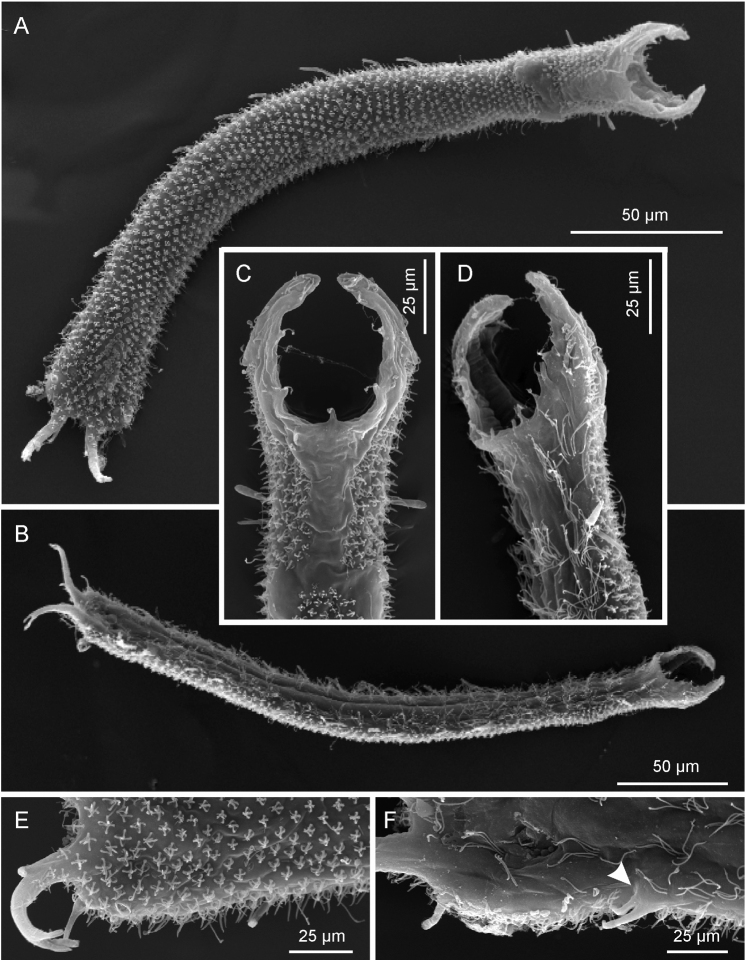
*Pseudostomella dolichopoda* sp. n. SEM photomicrographs. **A** habitus, dorsal view **B** habitus, ventral view; **C** close-up of the anterior region, dorsal view **D** close-up of the anterior region, ventrolateral view **E** close-up of the posterior region, dorsal view **F** close-up of the posterior region, ventral view, arrow shows the two ventrolateral adhesive tubes borne from a common base.

##### Material examined.

Fifteen adult specimens(including the holotype) collected by the author from different localities (see below); ten specimens were observed alive and are not longer extant, while five were prepared for SEM survey and are kept in the meiofauna collection of the author (Ref. n. 2002-BR-01-05).

##### Ecology.

Frequency of occurrence – common in sediment of the northern sites, usual in southern sites but occasional in locations facing the São Sebastião channel and on Ilhabela. Abundance - prevalent to numerous in sub-littoral sediment, scarce in littoral sediments where found.

##### Diagnosis.

A *Pseudostomella* with an adult length to 425 μm; pharynx length to 90 μm, with pharyngeal pores at base. Pharyngeo-intestinal junction (PhIJ) at U32; body slender, with graceful lines and elongate, furcate caudum. Head with mid-sized fleshy preoral palps curving around forward; palps showing few sensory hairs and provided with 5 and 8 papillae on the dorsal and ventral border respectively. Sensory hair sparce but evenly spaced on the body, forming lateral columns from about U12.5 to U85; glands barely visible, asymmetrically scattered along most of the length of the body. Cuticular armature of small, delicate pentancres on whole dorsal and ventrolateral surface, except for a bare, roughly T-shaped area posterior to the palps. Adhesive tubes: TbA, 2 per side in a row U7.8; TbDL, 1 per side, robust, inserting on lateral margin of the posterior trunk region at U83.5; TbVL, 11 per side, 2 smaller ones in the anterior pharyngeal region, roughly at U16, 7 of slightly variable size, irregularly spaced in the intestinal region from U33.5 to U71.5, the remainder 2 originate from a common base at U76.2; TbP, 4 per side, 2 + 1 at the end of each foot of the furcated caudum and the other one flanking each foot medially. Ventral locomotor cilia: a continuous field of transverse rows covering sparsely the entire surface from U12 to U35; the field splits in the anterior intestinal region to form paired lateral tufts that extend onto the ano-genital area at U84. Reproductive system: testis on the right body side, caudal organ inverted pyriform, at U78; frontal organ bladder-like, at U74.5; maturing eggs mid-dorsally above the posterior half of the intestine.

##### Etymology.

The specific name alludes to the extraordinary length of the caudal pedicles (*dólicos* Gr., long, and *poús*, *podos* Gr., foot).

##### Description.

The description is mainly based on the adult specimen, 358 µm in total length, shown in Figure 3. Body somewhat slender, little swollen in the posterior pharyngeal region and at the base of the 43 µm-long caudal pedicles. Pharynx 81 µm in length, measured from the ventral border of the oral opening to the pharyngeo-intestinal junction; pharyngeal pores near the base, at U29.5; pharyngeo-intestinal junction at U32; widths of neck\PhIJ\trunk\caudal base 30\29.5\40\31 µm at U15\U31\U51\U82, respectively. Head with well developed, fleshy preoral palps, incurving ventromedially; the dorsal border projecting just beyond the ventral. Sensory hairs and papillae occur on dorsal and ventral borders of the preoral palps; hairs are scattered on the dorsal, lateral and ventral surface of the palps; dorsally there are five papillae, nearly same in length (8–10 µm), symmetrically arranged along the inner border of the palps in a 2 + 1 + 2 pattern; ventrally, there are eight papillae, 5–8 µm in length, symmetrically arranged more centrally about the inner border of the palps in a 4 + 4 pattern; all papillae bearing one or two, short sensory hairs at their tip; other hairs form lateral columns that are evenly spaced from U12.5 to U85; individual hairs are 12–15 µm in length. Glands barely visible, variable in shape (oval to oblong) and size (4-8 µm in diameter), asymmetrically scattered along most of the length of the body.

*Cuticular armature*. Small sized pentancres with delicate, curved grasping tines, as tall as wide (3 × 3 µm – 5 × 5 µm) on whole dorsal and ventrolateral surface, except for a bare, roughly T-shaped area posterior to the palps; posteriorly most ancres extend onto the caudum.

*Adhesive tubes*. TbA, 2 per side (7-8 µm in length) in a row at U7.8; TbDL, 1 per side (15-18 µm in length), robust, inserting on the lateral margin of the posterior trunk region at U83.5; TbVL, 11 per side, 2 smaller ones (9-11 µm in length) in the anterior pharyngeal region, roughly at U16, 7 of slightly variable size and length (11-16 µm in length), irregularly spaced in the intestinal region from U33.5 to U71.5, the remainder 2, of unequal length (10 and 14 µm), originate from a common base at U76.2; TbP, 4 per side, 2 + 1 (5-6 µm in length) at the end of each foot of the furcated caudum and the other one (10 µm in length) flanking each foot medially.

*Ventral locomotor cilia*. A continuous field of transverse rows covering sparsely the entire surface from U12 to U35; the field splits in the anterior intestinal region to form paired lateral tufts that extend onto the ano-genital area at U84. Reproductive system: testis on the right body side, caudal organ inverted pyriform (11 × 22 µm), at U78; frontal organ bladder-like (9 µm in diameter), at U74.5; maturing eggs mid-dorsally above the posterior half of the intestine.

*Measurement and variability*. Body length of 15 living specimens ranged from 340-425 µm (mean = 388 µm, SD = 25.4 µm) all of them were mature (i.e., showed at least the testicles filled with sperm). Strange enough all of the SEM prepared specimens resulted of smaller size (i.e., less then 300 µm) even though only specimens appearing larger under the dissecting microscope were selected for this scope; these measurements are well below the 5.5% length reduction allowed for fixed specimens (cf. Clausen 2004a). The adhesive tubes of the TbVL series showed some variability in number, depending on individuals, ranging 11-15; however, the one borne on a common base numbered invariably two per side. Three specimens, 415-425 µm in total body length, collected in September 2003 from praia do Tenório and praia Grande showed 3-4 tubes of “cirrata” type along each dorsolateral side of the trunk region; cirrata tubes where never observed in specimens collected during the 2002 campaign. The meaning of these differences is unknown.

##### Taxonomic affinities.

Prior to the present study, the total number of *Pseudostomella* species known was 15 including one described by Valbonesi and Luporini (1984) but not formally named (cf. Hummon and Todaro 2010). Records come from a variety of locations including India and the Andaman Sea (e.g., Rao 1970, Rao 1993, Priyalakshmi et al. 2007); east Malaysia (Renaud-Mornant 1967); Somalia (Valbonesi and Loporini 1984); Portugal, Atlantic coast of France and north Sea (e.g., Swedmark 1956, Clausen 2004b, Hummon 2008); Mediterranean Sea (e.g., Hummon et al. 1993, Todaro et al. 2003); Atlantic and Gulf of Mexico coasts of the US (Ruppert 1970, Todaro et al. 1995); moreover, the recent addition of new taxa from Australia (Hochberg 2002), South Korea (Lee and Chang 2002) and South Africa (Todaro et al. 2011) testify the cosmopolitan distribution of the genus. By contrast, *Pseudostomella* species appear to have a relatively restricted geographic range, at least compared to the wide distribution or even the cosmopolitan nature of many other gastrotrichs (cf. Todaro et al. 1996, Curini-Galletti et al. 2012, Kanneby et al. 2012, Kieneke et al. 2012).

Gastrotricha Thaumastodermatidae, including species of *Pseudostomella*, are characterized by peculiar cuticular armatures made up of sculptured plates, spines or a combination of both. Species of the genera *Acanthodasys*, *Pseudostomella*, *Tetranchyroderma* and *Thaumastoderma* bear peculiar pronged scales called ancres: uniancres, triacres, tetrancres and pentancres depending on number of prongs.

*Acanthodasy* and *Thaumastoderma* species bear ancres of a single type only, uniancres and tetrancres respectively whereas *Pseudostomella* and *Tetranchyroderma* bear tri- tetra- or pentancres depending on species. In the latter two taxa the type of pronged cuticular armature has been regarded as the single most important taxonomic trait to classify species (e.g., Todaro 2002; Lee and Chang 2002, but see Todaro et al. 2011); consequently, within the genus *Pseudostomella* three basic species groups are envisioned based on type of pronged spines i.e., species characterized by triancres: *Pseudostomella faroensis* Clausen, 2004, *Pseudostomella klauserae* Hochberg, 2002, *Pseudostomella megapalpator* Hochberg, 2002, *Pseudostomella plumosa* Ruppert, 1970 and *Pseudostomella triancra* Hummon, 2011 (5 species). Forms that bear tetrancres: *Pseudostomella andamanica* Rao, 1993, *Pseudostomella indica* Rao, 1970, *Pseudostomella koreana* Lee & Chang, 2002, *Pseudostomella longifurca* Lee & Chang, 2002, *Pseudostomella malayica* Renaud-Mornant, 1967 and *Pseudostomella roscovita* Swedmark, 1956 (six species). Taxa that possess pentancres: *Pseudostomella cataphracta* Ruppert, 1970, *Pseudostomella cheraensis* Priyalakshmi, Menon & Todaro, 2007, *Pseudostomella etrusca* Hummon, Todaro & Tongiorgi, 1993, and *Pseudostomella* sp1 of Valbonesi and Luporini (1984) (four species).

Based on the type of ancres, the new species approaches the latter four taxa. However, *Pseudostomella cataphracta*, is unique in that it shows a group of four ventral adhesive tubes per side that is missing in the other species, while *Pseudostomella etrusca* is peculiar in thatit possesses a robust dorsal adhesive tubes inserted at base of each oral palp, a trait lacking in the other taxa. *Pseudostomella dolichopoda* sp. n. differs from *Pseudostomella cheraensis* mainly in virtue of its larger size (up 425 µm vs. up to 295 µm), presence of two adhesive tubes in the anterior pharyngeal region and on the presence of two adhesive tubes originated from a common base located in the posterior intestinal region; finally, *Pseudostomella dolichopoda* sp. n. differs from *Pseudostomella* sp1 because, among others, it has larger size (425 µm vs. 350 µm), possesses longer caudal pedicles (43 µm vs. 23 µm), bears two pairs of anterior adhesive tubes vs. a single pair present of Somali species, and because of the presence of the two adhesive tubes originate from a common base located in the posterior intestinal region.

##### Taxonomic key.

Hochberg (2002) and Lee and Chang (2002) in describing their new taxa, two species each, provided useful taxonomic keys; however, because two species were omitted in their analyses (i.e., *Pseudostomella andamanica* Rao, 1993 omitted in Hochberg, 2002 and *Pseudostomella* sp. 1 Valbonesi & Luporini, 1984 omitted in Chang and Lee 2002) and other taxa have been described in the meanwhile (see above), a revised key seems necessary. The key will hopefully prove useful not only to gastrotrich specialists but also to marine ecologists who find these peculiar metazoans in the course of research on interstitial meiobenthos.

**Table:** 

1	Cuticular armature of triancres	2
–	other	6
2	Five dorsal papillae on the prebuccal apparatus	3
–	seven dorsal papillae on the prebuccal apparatus	4
3	Four TbA per side; foot-like TbV present (3 tubes per side)	*Pseudostomella megapalpator* Hochberg, 2002
–	five TbA per side; foot-like TbV absent	*Pseudostomella klauserae* Hochberg, 2002
4	Scales of a triancre arise from a common, forked shaft	*Pseudostomella plumosa* Ruppert, 1970
–	each scale shaft arises independently from the base	5
5	Scales of triancres foliate (=scaled triancres	*Pseudostomella faroensis* Clausen, 2004
–	scales of triancres needle-like	*Pseudostomella triancra* Hummon, 2008
6	Cuticular armature of tetrancres	7
–	cuticular armature of pentancres	12
7	Five dorsal papillae on the prebuccal apparatus	8
–	seven dorsal papillae on the prebuccal apparatus	9
8	Five TbA per side; pedicles of 7 tubes (0:3:4); copulatory organ pyriform; bare area on the dorsal side, posterior to the prebuccal apparatus present	*Pseudostomella longifurca* Chang & Lee, 2002
–	two TbA per side; pedicles of 5 tubes (1:3:1); bare area absent	*Pseudostomella indica* Rao, 1970
9	copulatory organ tube-like	*Pseudostomella koreana* Chang & Lee, 2002
–	copulatory organ pyriform	10
10	Bare area on the dorsal side, posterior to the prebuccal apparatus present	*Pseudostomella roscovita* Swedmark, 1956
–	Bare area absent	11
11	TbL, 3 pairs; body short (about 200 µm in lenght)	*Pseudostomella malayica* Renaud-Mornant, 1967
–	TbL, 8 pairs; body elongate (about 500 µm in length)	*Pseudostomella andamanica* Rao, 1993
12	Five dorsal papillae	13
–	seven dorsal papillae; 3 TbA per side; pedicles of 5 tubes (1:3:1) each; foot-like TbV present (4 tubes per side)	*Pseudostomella cataphracta* Ruppert, 1970
13	Four TbA; single dorsal tube protruding from base of preoral palps present	*Pseudostomella etrusca* Hummon, Todaro & Tongiorgi, 1993
–	Two TbA; dorsal tube absent	14
14	Adhesive tubes along the pharyngeal region absent	*Pseudostomella cheraensis* Priyalakshmi, Menon & Todaro, 2007
–	Adhesive tubes along the pharyngeal region present	15
15	Single pair of adhesive tubes along the pharyngeal region; dorsal cuticular covering complete..	*Pseudostomella* sp.1 [Valbonesi & Luporini, 1984]
–	Two pairs of adhesive tubes along the pharyngeal region; presence of a bare area on the dorsal side, posterior to the prebuccal apparatus	*Pseudostomella dolichopoda* sp. n.

## Supplementary Material

XML Treatment for Pseudostomella dolichopoda
